# Oscillatory shear potentiates latent TGF-β1 activation more than steady shear as demonstrated by a novel force generator

**DOI:** 10.1038/s41598-019-42302-x

**Published:** 2019-04-15

**Authors:** Karim Kouzbari, Mohammad R. Hossan, Julien H. Arrizabalaga, Rohan Varshney, Aaron D. Simmons, Sandra Gostynska, Matthias U. Nollert, Jasimuddin Ahamed

**Affiliations:** 1 Cardiovascular Biology Research Program, Oklahoma Medical Research Foundation (OMRF), Oklahoma City, USA; 2 Department of Engineering and Physics, University of Central Oklahoma, Edmond, OK USA; 3 School of Chemical, Biological and Materials Engineering, University of Oklahoma, Norman, OK USA

## Abstract

Cardiovascular mechanical stresses trigger physiological and pathological cellular reactions including secretion of Transforming Growth Factor β1 ubiquitously in a latent form (LTGF-β1). While complex shear stresses can activate LTGF-β1, the mechanisms underlying LTGF-β1 activation remain unclear. We hypothesized that different types of shear stress differentially activate LTGF-β1. We designed a custom-built cone-and-plate device to generate steady shear (SS) forces, which are physiologic, or oscillatory shear (OSS) forces characteristic of pathologic states, by abruptly changing rotation directions. We then measured LTGF-β1 activation in platelet releasates. We modeled and measured flow profile changes between SS and OSS by computational fluid dynamics (CFD) simulations. We found a spike in shear rate during abrupt changes in rotation direction. OSS activated TGF-β1 levels significantly more than SS at all shear rates. OSS altered oxidation of free thiols to form more high molecular weight protein complex(es) than SS, a potential mechanism of shear-dependent LTGF-β1 activation. Increasing viscosity in platelet releasates produced higher shear stress and higher LTGF-β1 activation. OSS-generated active TGF-β1 stimulated higher pSmad2 signaling and endothelial to mesenchymal transition (EndoMT)-related genes PAI-1, collagen, and periostin expression in endothelial cells. Overall, our data suggest variable TGF-β1 activation and signaling occurs with competing blood flow patterns in the vasculature to generate complex shear stress, which activates higher levels of TGF-β1 to drive vascular remodeling.

## Introduction

The movement of blood in the mammalian cardiovascular system causes complex mechanical stress on both circulating blood cells, as well as on endothelial cells lining the vascular wall^1^. However, the cellular shear stress sensors and their role in regulating biological and pathological processes have not been clearly defined. Shear stress in the vasculature is a function of the detailed vasculature composition and blood flow rates^2^. So, the local shear rate is highly dependent on position and time. Shear is directly proportional to blood flow and inversely proportional to blood vessel diameter^3^. Altered shear stress is associated with several diseases, such as atherosclerosis and aortic stenosis, where narrowing of blood vessels and aortic valves obstructs blood flow^2,4–6^. These conditions can change shear patterns from normal physiological steady shear (SS) to higher and more complex oscillatory shear (OSS), depending on the wall structure and flow pattern. For example, OSS occurs at branched vessels, which are pro-atherogenic regions^7,8^, whereas laminar or SS occurs in unbranched unrestricted vessels, known to have anti-atherogenic effects^8,9^.

Cone-and-plate viscometers and flow chamber devices are traditionally used to quantify and analyze shear responses on cells and fluids, which varies with flow patterns and parameters such as cone angle, angular velocity, and gap height between cone tip and plate^10^. The cone-and-plate apparatus^11,12^ has been used to study the effects of laminar or turbulent fluid shear stress on cells and the relative contribution of fluid viscosity, time of exposure, and other physiological variables. It was found that OSS can produce differential effects on endothelial physiology including critical gene expression, cell proliferation, junction tightness, and permeability^13^. Although classical oscillatory flow produces a uniform sine wave when cone rotation is switched gently to the opposite direction, the mechanism and/or factors produced by OSS that underlies the different effects on cells are not known.

Transforming Growth Factor β1 (TGF-β1) is a potent and versatile cytokine. When activated, it exerts both physiologic and pathologic functions, including immune functions, inflammation, wound healing, and organ fibrosis^14^. All cells produce TGF-β1 in a latent complex (LTGF-β1) that requires activation to exert its biological function^15^. However, the mechanisms for LTGF-β1 activation remain unclear. Platelets contain 40–100-times more LTGF-β1 than any other cell type in the body^15^. We previously showed that various methods of generating complex shear forces, including stirring and vortexing, can induce LTGF-β1 activation partially via thiol disulfide exchange mechanism^16^. But, these methods produce a mixture of SS and OSS. We hypothesized that SS and OSS differentially activate LTGF-β1 and facilitate different biological effects. Since platelets are the major source of circulating TGF-β1, we further speculated that platelet-derived LTGF-β1 activated by shear directly affects endothelial cell biology, including endothelial to mesenchymal transition (EndoMT), a hallmark of excessive TGF-β1 activation and signaling^17^.

In this study, we programmed a cone-and-plate device to generate a spike in shear rate change during OSS by abruptly switching the cone rotation and modeled the change in shear rate by computational fluid dynamics (CFD) simulation to compare platelet-derived LTGF-β1 activation and its signaling responses in endothelial cells.

## Results

### Abrupt cone rotation generates a shear rate spike measured by computational fluid dynamics (CFD) simulation

**Figure 1:**
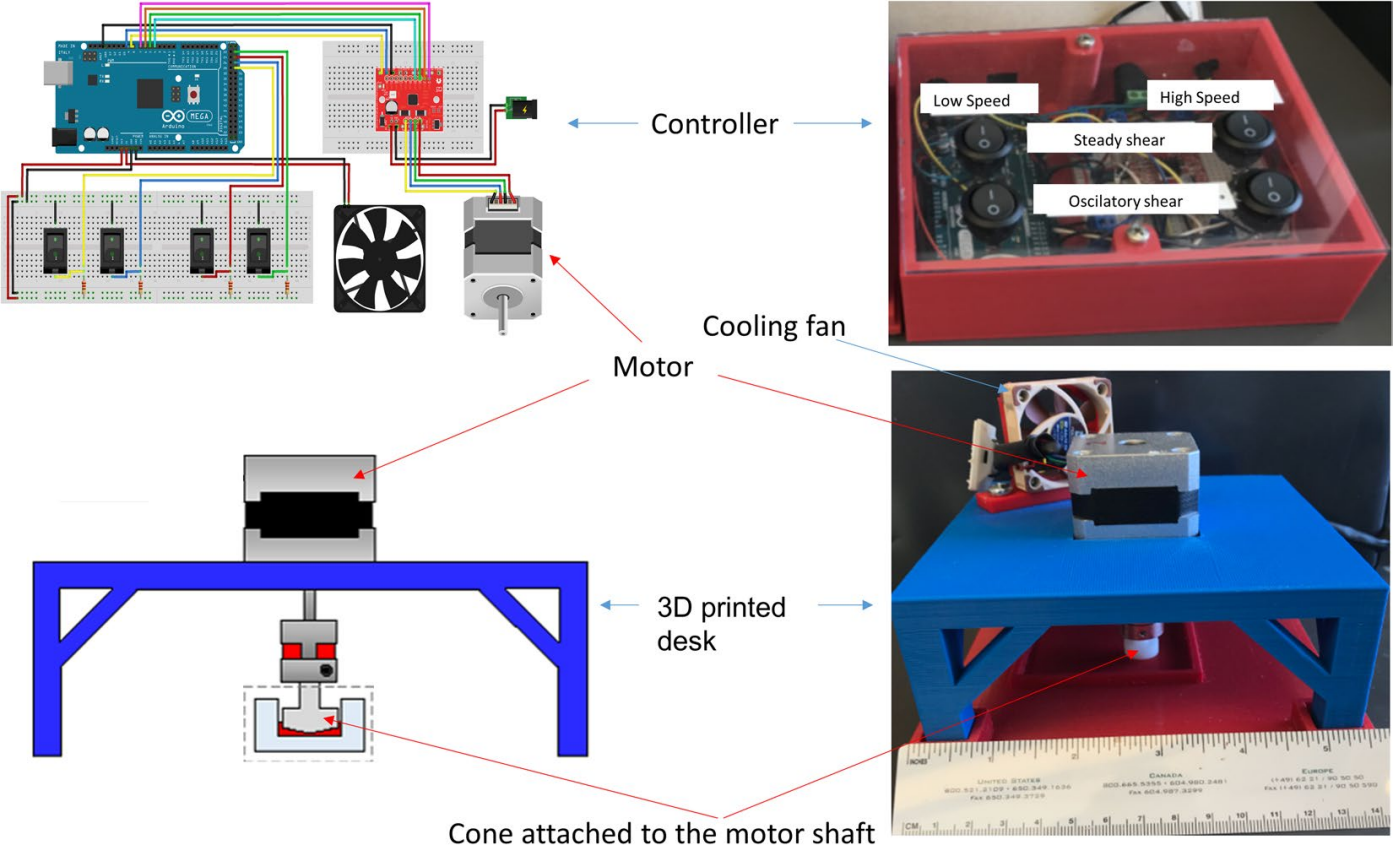
Design and picture of a cone-and-plate shear device that can generate both SS and OSS. Wiring diagram was drawn using Fritzing.org (developed by Friends-of-Fritzing, Germany) for the Arduino MEGA microcontroller connected to a Big Easy Driver stepper motor controller, with the ON/OFF rocker switches programmed to run either high or low speed and/or unidirectional to generate SS or switch directions to generate OSS (upper panels). The device consists of Delrin acetal resin cone connected via an aluminum shaft coupler to a stepper motor mounted on a custom-designed 3D printed mounting base (lower panels).

**Figure 2:**
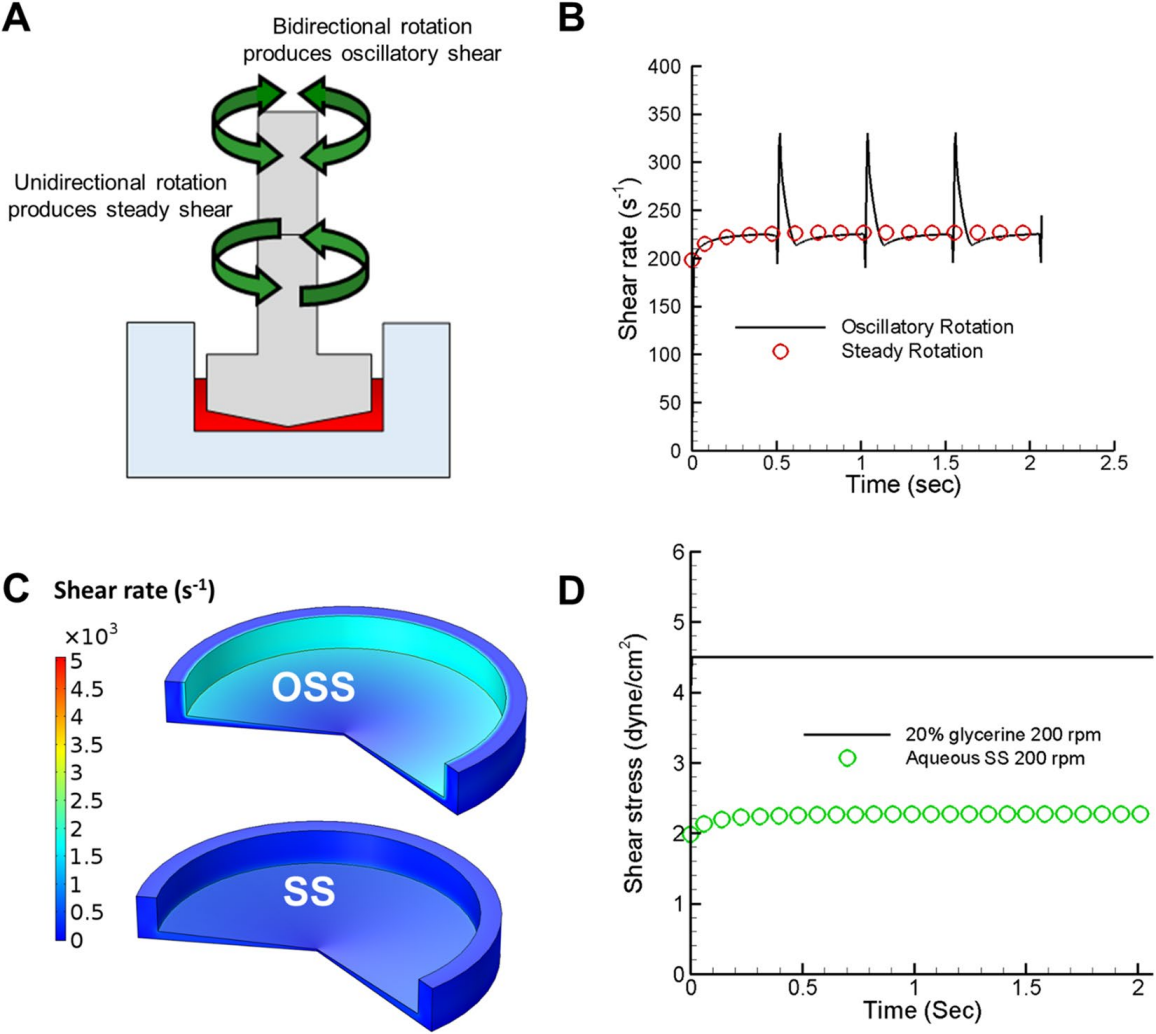
Computational modeling of SS vs. OSS in cone-and-plate containing platelet releasate suspension. **(A**) Schematic of the cone and plate with arrows pointing to unidirectional clockwise (lower arrows) and bidirectional (upper arrows) rotation, and the platelet releasate on the plate shown in red. (**B**) The volume average shear rate over time for a two complete bidirectional rotational cycles of the cone for a total 2.07 seconds. The computer simulation result shows the spike of shear rate change during the switch of direction transition time of 10 milliseconds at 200 rpm, red circle shows SS rate. (**C**) Computer simulation shows shear rate contour plot difference during OSS at time, t = 0.51 s and at unidirectional continuous SS mode. (**D**) The computer simulation result shows the increase in shear stress due to increased viscosity by adding 20% glycerol to the platelet releasate at 200 rpm.

We previously demonstrated that stirring and vortexing, which generates a complex shear environment consisting of both SS and OSS, activated LTGF-β1 in platelet releasates. We now sought to determine whether defined SS or OSS can differentially activate LTGF-β1. We programmed a custom-built cone-and-plate device capable of generating SS and OSS separately (Fig. 1). We generated SS by unidirectional continuous cone rotation or OSS using bidirectional switching of cone rotation abruptly for a 0.5 seconds cycle in each direction (Fig. 2A, and Video 1). Volume-averaged shear rate as a function of time for two complete cycles of the bidirectional rotation as well as steady unidirectional rotation was calculated by CFD at 200 rpm or 500 rpm. Figure 2B shows volume-averaged shear rate as a function of time for SS and OSS at 200 rpm. The shear rate was a smooth line throughout in unidirectional SS mode, while shear rate for the bidirectional OSS mode had repeated spikes in shear rate during rotational direction changes. The spike in shear rate during OSS was about 1.5 times the shear rate during SS (220 s^−1^ vs. 340 s^−1^). The overall shear rate distribution in the cone-and-plate device at time t = 0.51 s during transition of rotational direction change is also higher (Fig. 2C). The maximum shear rate is observed near the surface of the cone in the both SS and OSS mode. CFD simulation showed increasing viscosity with 20% glycerol caused >2-fold higher shear stress than buffer at 200 rpm (Fig. 2D).

### LTGF-β1 activation in platelet releasates under SS and OSS

**Figure 3:**
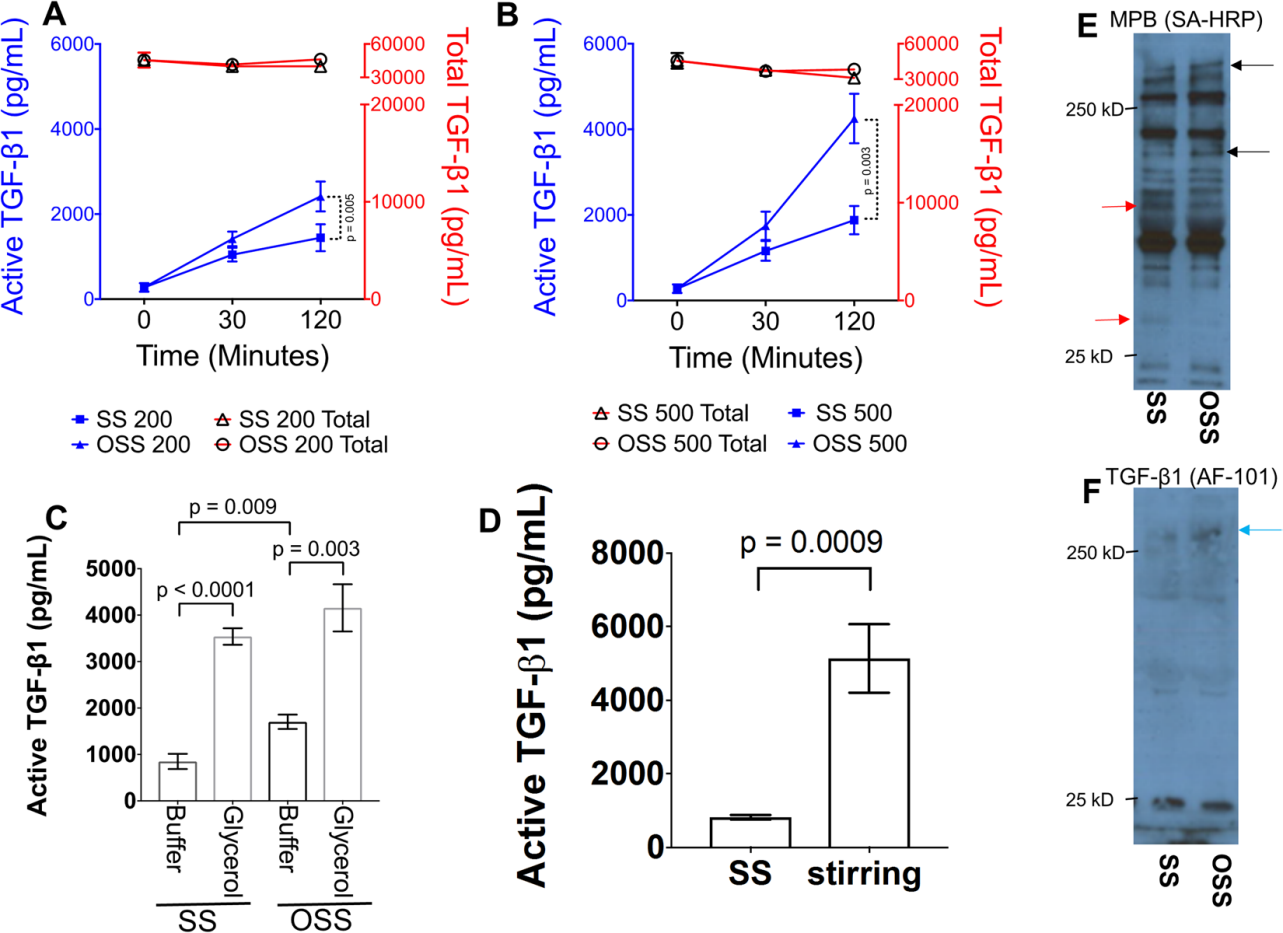
OSS induces higher LTGF-β1 activation than SS. (**A**,**B**) Platelet releasates were incubated in the cone-and-plate device with SS or OSS at either 200 or 500 rpm for 30 and 120 minutes. Active TGF-β1 was measured directly in the ELISA (blue-color), whereas total TGF-β1 (latent + active) was measured after activating latent TGF-β1 by treatment with acid (red color). (**C**) Increasing viscosity in platelet releasates by adding 20% glycerol, dramatically increased the active TGF-β1. (**D**) Platelet releasates were sheared at 200 rpm SS condition in cone-and-plate device or stirred in a 96 well plate with stirring bar at 200 rpm. Active TGF-β1 was measured by ELISA (n = 5–6). Error bars represent SD. (**E**) Platelet releasates were SS or OSS for 120 min and then labeled with MPB (100 μm) for 20 min. MPB-labeled proteins were identified with streptavidin (SA) HRP. Black arrows indicate protein bands with increased MPB labeling, and red arrows indicate protein band with decreased MPB labeling. (**F**) Same samples were immunoblotted with anti-TGF-β1 antibody (AF-101-NA), mature TGF-β1 band migrates at ~25 kD band, whereas a HMW band (>250 kD) appears in OSS (blue arrow).

To test the effects of SS vs. OSS on LTGF-β1 activation, we subjected platelet releasates to either SS or OSS at different shear rates for varying exposure times. We found that OSS generated significantly higher levels of active TGF-β1 in a time- and dose-dependent manner than samples subjected to SS (Fig. 3A,B). Active TGF-β1 levels increased from 1445 ± 842 to 2416 ± 994 pg/ml at 200 rpm (p = 0.005) and from 2030 ± 1052 to 4252 ± 1632 pg/ml at 500 rpm (p = 0.003) after 2 hours in SS vs. OSS, respectively. No significant change in total TGF-β1 levels were observed in any shear conditions (Fig. 3A,B). Increasing viscosity in platelet releasates produced higher TGF-β1 activation than observed in buffer during SS (Fig. 3C). This is consistent with the higher shear stress computed in viscous platelet releasate (Fig. 2D). We compared LTGF-β1 activation by stirring and found that stirring generated higher active TGF-β1 than SS (4250 ± 1004 pg/ml in stirring vs. 1445 ± 842 pg/ml in SS; p = 0.009) (Fig. 3D).

Our previous observations indicated a loss of free thiols in platelet releasate proteins, including TGF-β1 and thrombospondin 1 upon stirring as assayed by decreased MPB labeling and mass spectrometry^16,18^. We therefore now examined potential differences in thiol labeling between SS vs. OSS. Incubation of platelet releasates with OSS, altered the thiol labeling pattern of proteins with MPB. Some proteins showed increased labeling (*black arrows*), including high molecular weight (HMW) protein, while others showed reduced labeling (*red arrows*) (Fig. 3E). We also observed an accumulation of HMW protein complex(es) (>250 kDa, band(s), correcponding to MPB-labelled band, also immunoblotted with anti-TGF-β1 antibody (Fig. 3F). These bands disappear under reducing condition (data not shown). These data indicate higher thiol-disulfide exchange appear in OSS than SS, leading to oxidation free thiols resulting disulfide bonded HMW complex formation.

### OSS-generated active TGF-β1 stimulates higher Smad2 phosphorylation

**Figure 4:**
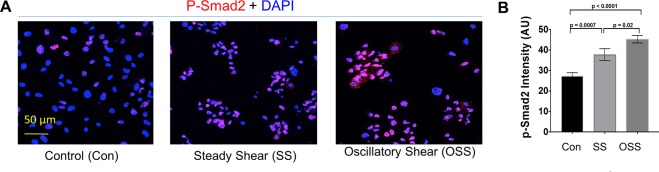
OSS-activated platelet releasate induces higher Smad2 phosphorylation (P-Smad2) than SS-activated samples. Endothelial cells (HUVECs) incubated with OSS or SS or unsheared (Con) platelet releasates for 6 hours. Cells were fixed, permeabilized, and stained with p-Smad2-specific antibody. Nucleus was counterstained with DAPI shown in blue. P-Smad2 intensity in the nucleus was quantified using ImageJ, and intensity was plotted in arbitrary units (AU) (n = 4–6).

To test whether different levels of active TGF-β1 generated by SS and OSS differentially induce cellular signaling, we incubated human umbilical vein endothelial cells (HUVEC) with SS- or OSS-activated platelet releasates for 6 hours, which induced phosphorylation of Smad2 (P-Smad2) signaling as measured by immunofluorescence staining. P-Smad2 staining intensity in the nucleus was higher in OSS- than SS-stimulated cells (Fig. 4A,B). These data indicate higher active TGF-β1 generated by OSS stimulated more nuclear p-Smad2 signaling in endothelial cells.

### OSS-induced active TGF-β1 stimulates higher EndoMT markers, PAI-1, collagen, and periostin expression in endothelial cells

**Figure 5:**
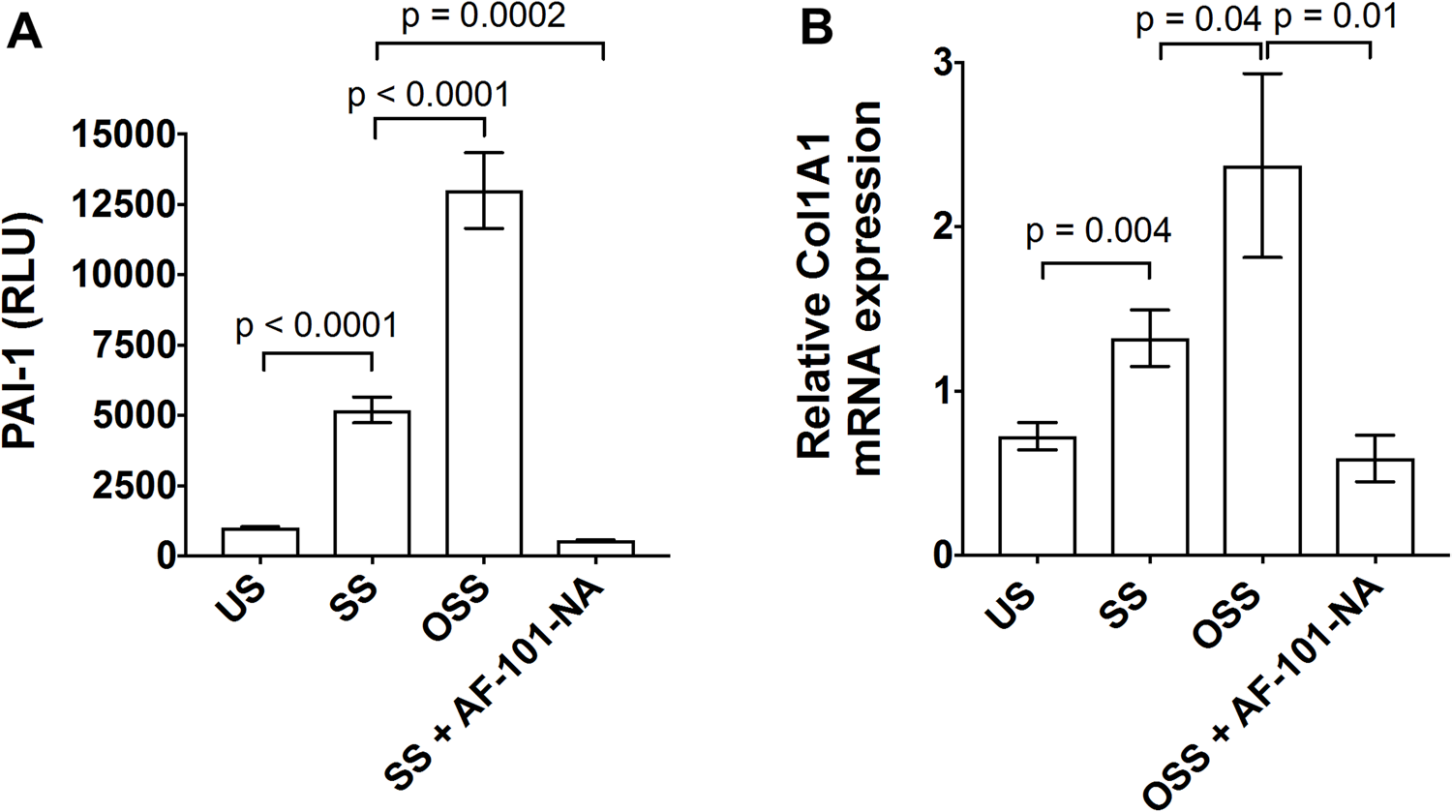
OSS-activated TGF-β1 stimulated PAI-1 luciferase activity in MLEC and collagen expression in endothelial cells. (**A**) MLECs were stimulated with OSS or SS or unsheared (US) platelet releasates for 18 hours with and without anti-TGF-β1 neutralizing antibody (AF-101-NA). PAI-1 luciferase activity was measured using luminometer and data were plotted as relative luminescence unit (RLU). (**B**) HUVECs were stimulated with OSS- or SS-activated platelet releasates or unsheared platelet releasates (US) with and without anti-TGF-β1 antibody for 6 hours, collagen (*Col1a1*) gene expression was measured by RT-PCR (n = 3–4).

Active TGF-β1 signaling induces endothelial to mesenchymal transition (EndoMT), assessed by a series of TGF-β1-responsive gene programs, including PAI-1 and collagen expression. To determine whether OSS-generated active TGF-β1 can induce higher PAI-1 induction, we used an MLEC cell-based PAI-1 luciferase assay. MLECs were stimulated with platelet releasates subjected to 2 hours of SS or OSS. We found that platelet releasates subjected to OSS induced higher PAI-1 luciferase activity than SS-subjected releasates (Fig. 5A). We stimulated HUVECs for 6 hours with SS- vs. OSS-generated active TGF-β1 and found higher expression of *Col1a1* gene in OSS compared to SS samples (Fig. 5B). These responses were almost completely blocked by anti-TGF-β1 antibody, which showed the specificity of active TGF-β1-generated by OSS. We recently showed HUVEC cultured with the activated form of platelet-derived TGF-β1 induced EndoMT markers, α-SMA and vimentin^19^. In addition, incubation of HUVEC with OSS induced higher periostin expression than cells cultured with SS (data not shown). These results indicate that higher TGF-β1 activation by OSS can induce downstream signaling for EndoMT.

## Discussion

Our study shows that OSS generates biologically-active TGF-β1 at a significantly higher rate than SS. To delineate if OSS could lead to higher TGF-β1 activation than SS, we designed a cone-and-plate device that utilized precise control of rotation to generate a spike in shear stress during OSS, which resulted in higher LTGF-β1 activation than SS. One advantage of our device, which can generate both SS and OSS, is that it uses the same settings in the same and different samples to avoid inter- and intra-assay variables. This system has another advantage in that it can be incorporated in a 96-well manifold, enabling high-throughput screening of inhibitors/activators of sheared sensor molecule targets, such as LTGF-β1^16,18,20^ and von Willibrand factor (vWf)^21^. Thus, these modifications could prove advantageous for drug development for diseases involving high and complex shear, such as atherosclerosis^22^, aortic stenosis^6^, or lymphedema^23^, where changes in shear stress occur.

Our shear simulation with computer modeling demonstrates the striking difference in shear rate changes between SS and OSS. During the transition from one directional rotation (e.g. counter clockwise) to the other (e.g. clockwise), the cone decelerates to a brief stop, then accelerates to reach a steady rotational speed. The spike in the shear rate, as well as shear stress, occurs during this transition period. During the brief stops, although fluid adjacent to the cone-and-plate surface also stops, the fluid between the cone and the plate remains in motion due to inertia. Even in the acceleration phase after stopping, where fluid adjacent to the cone surface follows the rotational direction of cone, the fluid away from the cone surface rotates in the opposite direction, again due to inertia. Thus proteins in the fluid phase experience opposing shear rates as well as shear stress every 0.5 seconds, and the effects accumulate over time.

The higher molecular strain due to the change of shear rate during the abrupt switch of cone rotation (tangential interludes) highlights a critical factor for higher LTGF-β1 activation. However, the mechanism through which shear activates TGF-β1 requires further study to identify specific molecular shear sensor regions, such as the vWf A2 domain^24^. The crystal structure of TGF-β1 suggests it may be too small to sense shear, but whole latent complex with LTBP-1 structure may provide insight into these activation mechanisms^25^. We previously demonstrated that LTBP-1 in the latent complex is required for shear-dependent LTGF-β1 activation^16^. Our previous study also showed thiol disulfide exchange contributes to shear-dependent LTGF-β1activation. Our data indicates that OSS induces higher thiol oxidation and disulfide-bonded HMW complex(es) accumulation, confirming our previous implication that thiol disulfide exchanges contribute to shear-dependent LTGF-β1 activation^16,18,26^. It is possible that the LTGF-β1 is associated with disulfide-bonded complex(es) that is much larger than proteins like vWf, which can itself be sensed by shear. Future study is needed to elucidate additional mechanistic insight for LTGF-β1 activation by SS vs. OSS via thiol disulfide exchange mechanism.

Our findings have both physiological and pathological significance, as both SS and OSS are generated in different parts of the vasculature depending on the geometry. For example, areas exposed to unidirectional SS, considered non-pathological shear stress like non-obstructive areas in the common carotid artery, remain relatively healthy, while areas of bifurcation and other obstructive areas where blood flow is disturbed as in OSS are considered pathological as they are affected by higher deposition of atherosclerotic plaques. Studies showed that oscillatory shear stresses increase the inflammatory and thrombotic potential of endothelial cells^2,4^. Thus, low amounts of active TGF-β1 generated by SS may be beneficial as it is a known anti-inflammatory cytokine^27^. However, conditions, such as aortic stenosis that have increased shear through the narrowed aortic valve, generate OSS, leading to aortic valve stiffening due to fibrosis, a function of excessive TGF-β1 production and signaling.

We recently showed that shear-activated platelet TGF-β1 directly contributes to aortic stenosis (AS) progression in a robust mouse model^19^. EndoMT is a phenomenon induced by TGF-β1 signaling^17^, and we found platelets are physically attached with valvular endothelial cells inducing mesenchymal transition^19^. These results suggest further studies should determine whether OSS-induced TGF-β1 activation around valve leaflets contributes to AS progression. Our data shows that platelet releasates subjected to OSS can induce TGF-β signaling in endothelial cells for p-Smad2 and genes responsible for the EndoMT process, a hallmark of pathologic organ fibrosis^28^. These findings suggest that areas where OSS is generated, such as in aortic stenosis, are more likely to display EndoMT, a hallmark of atherosclerosis^29^ and fibrosis^17^.

Our data also suggest increased TGF-β1 activation in OSS may have clinical implication related to hemodynamic changes seen during atherosclerotic plaque rupture and subsequent thrombosis in myocardial infarctions, where high shear (turbulent/oscillatory) is generated by tissue factor-mediated thrombus formation and platelet activation inside the closing vessels^2,4^. During this event, platelet TGF-β1 activation may substantially induce signaling for fibrosis development later in life. Our study shows that higher TGF-β1 activation by OSS leads to increased TGF-β1 signaling as measured by p-Smad2, PAI-1, and collagen synthesis. These findings are consistent with a recent report showing that oscillatory shear exposure induces collagen deposition in fibrotic aortic valves^30^. We also posit that areas in which oscillatory shear occurs, such as AS and/or atherosclerotic plaques, may predict a deleterious outcome for fibrosis or plaque ruptures^1,31^. Thus, our results have pathological implications *in vivo* where similar measurements can calculate the effects of different forms of shear changes, such as remodeling during wound healing or pathological remodeling in fibrosis. Studies using *in vivo* animal models will validate this hypothesis.

In conclusion, our data demonstrate that OSS induces higher active TGF-β1 production and signaling, which may have distinct biological and pathological effects in vascular areas exposed to SS vs. OSS. Future studies should measure *in vivo* shear changes to correlate with vasculature remodeling, pathological fibrosis in conditions such as AS, atherosclerosis, aneurism, and coarctation-induced pressure overload models that simulate high shear conditions, which result in platelet activation and organ fibrosis. In addition, our new system may be useful to screen and validate inhibitors/activators for shear-sensitive molecules/cells/factors, such as LTGF-β1 activation and von Willibrand factor (vWf) cleavage, relevant to fibrosis, inflammation, thrombosis, and bleeding.

## Materials and Methods

### Materials

Antibodies and ELISA kits for TGF-β1 were obtained from R&D Systems and used as described previously^16,18,26,32^.

### Preparation of platelet releasate

Human studies were approved by the OMRF Institutional Review Board. After informed consent was obtained in accordance with the Declaration of Helsinki, blood was drawn from healthy volunteers using a 19-gauge needle and a syringe containing 0.38% citrate. In some cases, freshly isolated units of platelets were obtained from the Oklahoma Blood Institute (Oklahoma City, OK). Washed platelets (1 × 10^9^/mL) were prepared and stimulated with thrombin (0.125 U/mL) for 5 minutes at 37 °C as previously described^16^. Platelet releasates were prepared by centrifugation at 14,000 *g* for 20 minutes at 4 °C and assayed for active and total TGF-β1 as previously described^16^.

### Building a cone-and-plate shear device that generates both SS and OSS

We designed and built a cone-and-plate shear device capable of generating either steady shear (SS) or oscillatory shear (OSS) by rotation direction controlled by an Arduino MEGA System connected to a Big Easy Driver stepper motor controller with an ON/OFF rocker switch. The schematic of the design and the device are shown in Fig. 1. The cone is made of Delrin acetal resin to render it biocompatible, and has a 2.5 degree angle and a diameter of 14 mm^1^. The diameter of the plate is 15.8 mm, with a gap 0.9 mm between the cone and the plate on each side. The cone was connected to a stepper motor (bipolar, 200 steps/rev, NEMA 17 size) via an aluminum shaft coupling. The mounting base for the stepper motor was designed using Solidworks 2017 and 3D printed with poly-lactic acid (PLA) on a Makerbot Replicator 5^th^ generation. An A4988 Allegro-based micro stepping driver (Big Easy Driver) was supplied with 24 V DC and connected to an Arduino MEGA 2560-R3 microcontroller to control the stepper motor. The Arduino Integrated Development Environment (IDE; version 1.8.2) was used to program the microcontroller to switch the rotation mode between SS and OSS, by controlling the rotational velocity, and the period of oscillation/switch rotational direction. The device was programed to rotate the cone a full cycle for 0.5 seconds in either counter-clockwise or clockwise at a fixed speed of 200 or 500 rpm to generate SS. To generate OSS, the cone rotation was abruptly changed to the opposite direction with an intermittent 10 milliseconds (ms) stopping of the cone between the rotations of the cone a full cycle for 0.5 seconds (see Video 1A,B).

### Computational fluid dynamics (CFD) modeling for calculation of shear rate generated by SS and OSS

When the cone attached to motor rotated in a single direction, SS is generated, and when rotated in both directions, OSS is generated (Fig. 2A). The fluid flow patterns and shear rate in SS and during the transitions to OSS were calculated by computer simulation. Due to the rotational symmetry of the cone-and-plate device, we considered a two-dimensional axisymmetric vertical cross-section of the device for the computer simulation. The device was filled with incompressible and Newtonian fluid up to 2.1 mm height with a density of 1000 kg/m^3^ and a dynamic viscosity of 1e-3 Pa.s, which corresponds to 135 microliters of platelet releasate suspension, as used for our experiments. The transient Navier-Stokes equations in two-dimensional axisymmetric coordinates were solved using the finite element method in Multiphysics software COMSOL 5.2a.

The volume averaged shear rate was estimated from the shear rate of individual elements of the discretized cone-and-plate device. Two full cycles of bidirectional rotation were considered in the simulation of OSS. Acceleration and deceleration of the cone between the changes of rotational direction was achieved at 10 ms each through a linear piecewise function. Grid refinement analysis was performed to determine optimum mesh density at which the solution does not vary. All simulation results were performed with that optimum mesh density.

### Effect of shear (SS and OSS) on LTGF-β1 activation

Platelet releasates (250 μL) prepared as described above, were placed in a polystyrene plastic plate (Nalge-Nunc International, Roskilde, Denmark). The cone was placed on the plate and subjected to shear with SS or OSS modes using the device (Fig. 1) for up to 2 hours. Samples were collected at the indicated time points and assayed for active and total TGF-β1 levels. In some cases, the viscosity of platelet releasates was increased by adding 20% glycerol and then subjecting them to shear as indicated. In parallel, samples were incubated without shear and used as unsheared (US) controls. LTGF-β1 activation was compared by stirring platelet releasates (100 μL) at 200 rpm in a well on 96-well plate containing metal stir bars (5 mm) by magnetic stirrer for 2 hours and then assayed for active and total TGF-β1.

### Active TGF-β1 generated by SS and OSS stimulates cell signaling measured by cell-based assays

Endothelial cells (HUVECs from Lonza) were stimulated with SS- or OSS-activated TGF-β1 for 6 hours, Smad2 phosphorylation (p-Smad2) was measured by immunofluorescence. HUVECs were stimulated with samples activated by SS or OSS for 6 hours, total RNA was extracted from endothelial cells using a QIAGEN kit. cDNA was prepared from RNA using a High Capacity RNA-to-cDNA Kit (Applied Biosystems). Real-time PCR was performed with ready-made primer sets for *collagen type1 alpha1* (*Col1a1*) periostin (*Postn*) genes using a Biorad real time PCR system. HUVECs (Lonza) were stimulated with SS or OSS containing TGF-β1 for 24 hours, cells were washed, fixed, permeabilized, and stained with periostin antibody (Abcam). Mink lung epithelial cells (MLEC) expressing PAI-1 luciferase construct (kindly gifted by Dr. D. Rifkin, NYU, NY) were stimulated by samples activated by SS or OSS. PAI-1 luciferase activity was measured after ON stimulation by a luminometer. Data were expressed as relative luminescence unit (RLU).

### Statistical analysis

Data are expressed as mean plus or minus SD/SEM. The significance of differences was calculated by Student *t*-test using graphpad Prism-7.

## Supplementary information

OSS motion

SS motion

Images cropped from the Original Immunoblots
