# Supplementary figures and images for: Oscillatory shear potentiates latent TGF-β1 activation more than steady shear as demonstrated by a novel force generator
Source: Sci Rep. 2019 Apr 15;9:6065. doi: 10.1038/s41598-019-42302-x (PMC6465594; doi:10.1038/s41598-019-42302-x)

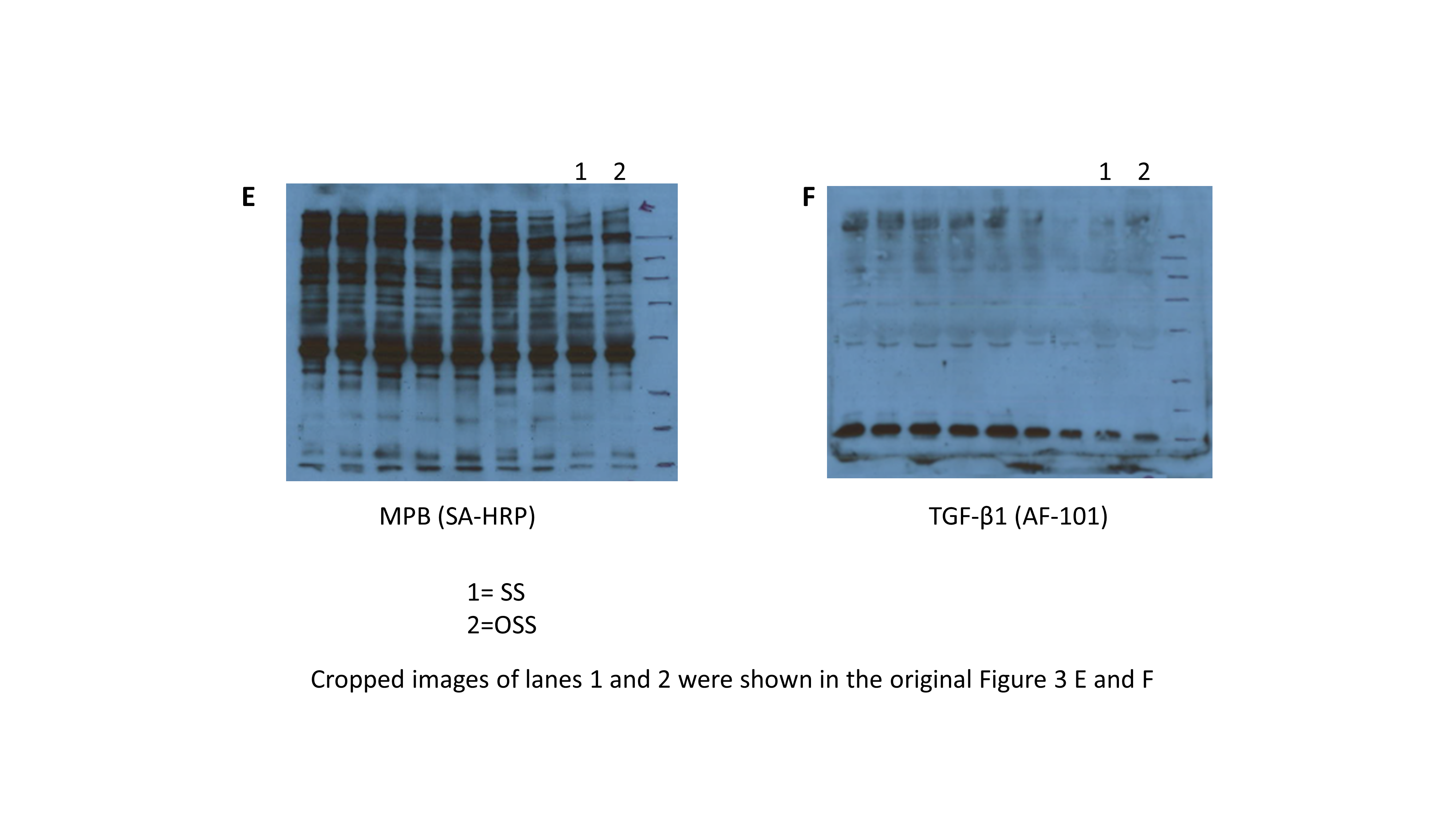

Supplement: Supplementary file 3 — Images cropped from the Original Immunoblots [file 41598_2019_42302_MOESM3_ESM.tif]
